# 2-((*E*)-{4-[Bis(4-eth­oxy­phen­yl)amino]­phen­yl}imino­meth­yl)phenol

**DOI:** 10.1107/S1600536814003201

**Published:** 2014-02-15

**Authors:** Bing-Fei Gao, Zhe-Peng Jin, Jiang Chen, Yu-Peng Tian

**Affiliations:** a Department of Chemistry, Anhui University, Hefei 230039, People’s Republic of China; b Key Laboratory of Functional Inorganic Materials Chemistry, Hefei 230039, People’s Republic of China; c Department of Chemistry, Bengbu Medical College, Bengbu 233030, People’s Republic of China

## Abstract

In the title Schiff base mol­ecule, C_29_H_28_N_2_O_3_, the three terminal benzene rings are twisted by 73.84 (15), 81.25 (16) and 12.1 (2)° with respect to the central benzene ring. An intra­molecular O—H⋯N hydrogen bond occurs. In the crystal, mol­ecules are linked *via* weak C—H⋯π inter­actions into a three-dimensional supra­molecular architecture.

## Related literature   

For background and the synthesis of the title compound, see: Dharmaraj *et al.* (2001 ▶); Feng (2014 ▶). For a related structure, see: Tanak *et al.* (2013 ▶).
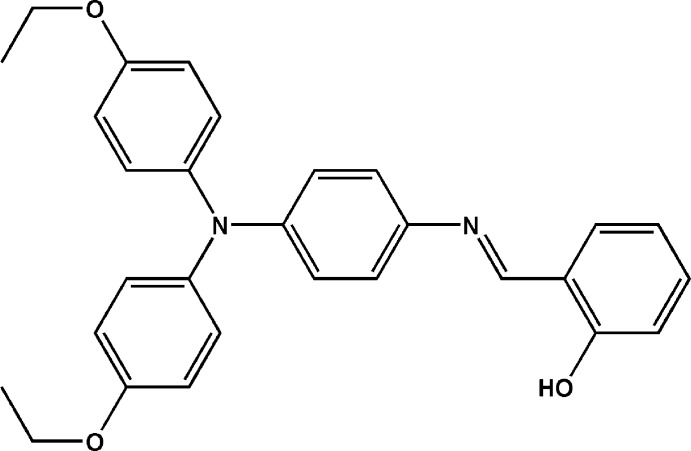


## Experimental   

### 

#### Crystal data  

C_29_H_28_N_2_O_3_
*M* *_r_* = 452.53
Orthorhombic, 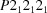

*a* = 9.765 (3) Å
*b* = 13.113 (4) Å
*c* = 19.378 (6) Å
*V* = 2481.2 (14) Å^3^
*Z* = 4
Mo *K*α radiation
μ = 0.08 mm^−1^
*T* = 296 K
0.40 × 0.30 × 0.20 mm

#### Data collection  

Bruker SMART CCD area-detector diffractometer
Absorption correction: multi-scan (*SADABS*; Bruker, 2001 ▶) *T*_min_ = 0.969, *T*_max_ = 0.984
17684 measured reflections
2486 independent reflections
1747 reflections with *I* > 2σ(*I*)
*R*_int_ = 0.051

#### Refinement  

*R*[*F* ^2^ > 2σ(*F* ^2^)] = 0.044
*wR*(*F* ^2^) = 0.113
*S* = 1.13
2486 reflections
310 parameters
6 restraints
H-atom parameters constrained
Δρ_max_ = 0.12 e Å^−3^
Δρ_min_ = −0.18 e Å^−3^

### 

Data collection: *SMART* (Bruker, 2007 ▶); cell refinement: *SAINT* (Bruker, 2007 ▶); data reduction: *SAINT*; program(s) used to solve structure: *SHELXS97* (Sheldrick, 2008 ▶); program(s) used to refine structure: *SHELXL97* (Sheldrick, 2008 ▶); molecular graphics: *SHELXTL* (Sheldrick, 2008 ▶); software used to prepare material for publication: *SHELXTL*.

## Supplementary Material

Crystal structure: contains datablock(s) I, Global. DOI: 10.1107/S1600536814003201/xu5765sup1.cif

Structure factors: contains datablock(s) I. DOI: 10.1107/S1600536814003201/xu5765Isup2.hkl

Supporting information file. DOI: 10.1107/S1600536814003201/xu5765Isup3.cml

CCDC reference: 986499

Additional supporting information:  crystallographic information; 3D view; checkCIF report

## Figures and Tables

**Table 1:** Hydrogen-bond geometry (Å, °) *Cg*1 *Cg*2 and *Cg*3 are the centroids of the C3–C8, C11–C16 and C17–C22 benzene rings, respectively.

*D*—H⋯*A*	*D*—H	H⋯*A*	*D*⋯*A*	*D*—H⋯*A*
O3—H3⋯N2	0.82	1.87	2.605 (4)	148
C1—H1*C*⋯*Cg*2^i^	0.96	2.84	3.760 (4)	162
C4—H4⋯*Cg*3^ii^	0.93	2.82	3.655 (3)	150
C9—H9*C*⋯*Cg*1^iii^	0.96	2.94	3.872 (4)	164
C28—H28⋯*Cg*1^iv^	0.93	2.88	3.742 (4)	154

Symmetry codes: (i) 
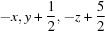
; (ii) 
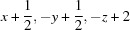
; (iii) 
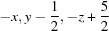
; (iv) 
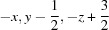
.

**Fig. 1.**
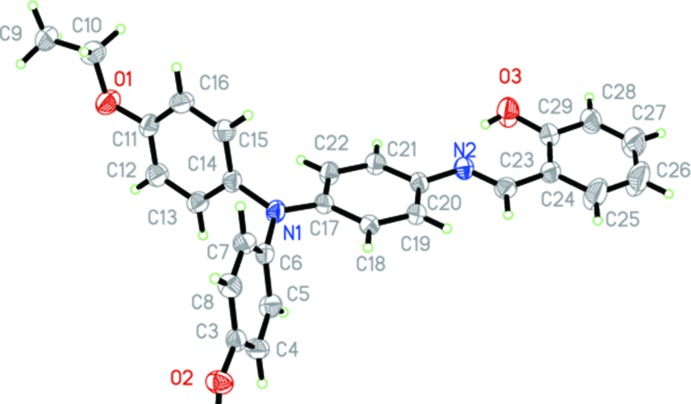
: The molecular structure of (I), with the atom-numbering scheme. Displacement ellipsoids are drawn at the 30% probability level.

**Fig. 2.**
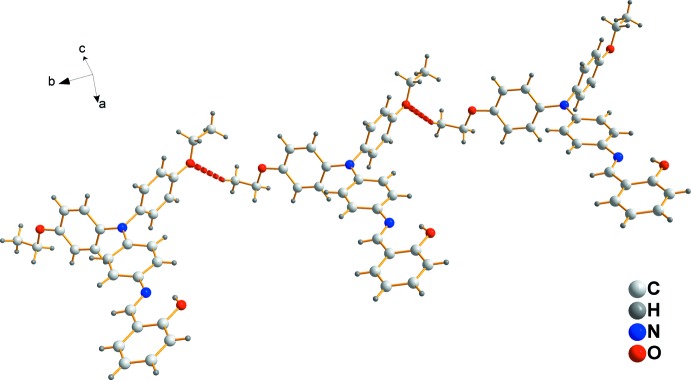
: The H-bond diagram of the title molecule(I).

## supplementary crystallographic information

### 1. Comment

In the past few decades, the researchers have witnessed a great deal of interest in the chemistry of transition metal Schiff base chelates bacause of their importance as catalysts in reactions such as carbonylation, hydroformylation, reduction, oxidation, epoxidation and hydrolysis (Dharmaraj *et al.*, 2001). Schiff bases derived from salicyladehyde and fluoroaniline, specifically, have been considered as potential pharmaceutically interesting compounds as several of the members of this family of molecules have shown antimicrobial, antitumor or antiviral activities (Feng *et al.*, 2014). As an extension of our work on the structural characterization of Schiff base compounds, the crystal structure of the title compound (I) is reported.

In (I) (Fig.1), the molecular structure of title compound shows an E configuration, with a C(20)—N(2)=C(23)—C(24) torsion angle of -176.9 (3)°. The bond distance of N(2)=C(23) at 1.258 (3) Å is a typical double bond. The dihedral angles between the salicyladehyde moiety and the aniline ring is 12.1 (2)°, indicating they are nearly coplanar. Intramolecular O—H···N hydrogen bond exists, similar to that found in a related structure (Tanak *et al.* 2013). In the crystal, the molecules are linked via weak C—H···π interaction into the three dimensional supramolecular architecture.

### 2. Experimental

A hot solution of N^1^,*N*^1^ -bis (4-ethoxyphenyl) benzene -1,4 - diamine (3.48 g, 10 mmol) in ethanol 50 mL was mixed with a salicylaldehyde (1.83 g, 15 mmol) in ethanol 5 mL, a yellow solid was appeared immediately, then the reaction mixture was reflux for 2 h. Under cooling the room temperature, the light yellow crystals were separated by filtration and recrystallized from ethanol. Yield: 96%. ^1^H NMR (400 MHz, DMSO-d^6^) 1.34 (t, 6H), 4.03 (m, 4H), 6.82 (d, 2H), 6.97 (m, 6H), 7.04 (d, 4H), 7.31 (d, 2H), 7.38 (t, 1H), 7.59 (d, 1H), 8.90 (s, 1H), 13.37 (s, 1H).

### 3. Refinement

All hydrogen atoms were placed in geometrically idealized positions and constrained to ride on their parent atoms, with C—H = 0.93 Å and *U*_iso_(H) = 1.2*U*_eq_(C). As no significant anomalous scattering, Friedel pairs were merged.

### Crystal data

**Table:**

C_29_H_28_N_2_O_3_	*D*_x_ = 1.211 Mg m^−^^3^
*M**_r_* = 452.53	Mo *K*α radiation, λ = 0.71073 Å
Orthorhombic, *P*2_1_2_1_2_1_	Cell parameters from 2336 reflections
*a* = 9.765 (3) Å	θ = 2.3–20.7°
*b* = 13.113 (4) Å	µ = 0.08 mm^−^^1^
*c* = 19.378 (6) Å	*T* = 296 K
*V* = 2481.2 (14) Å^3^	Block, yellow
*Z* = 4	0.40 × 0.30 × 0.20 mm
*F*(000) = 960	

### Data collection

**Table:**

Bruker SMART CCD area-detector diffractometer	2486 independent reflections
Radiation source: fine-focus sealed tube	1747 reflections with *I* > 2σ(*I*)
Graphite monochromator	*R*_int_ = 0.051
phi and ω scans	θ_max_ = 25.0°, θ_min_ = 1.9°
Absorption correction: multi-scan (*SADABS*; Bruker, 2001)	*h* = −11→11
*T*_min_ = 0.969, *T*_max_ = 0.984	*k* = −15→15
17684 measured reflections	*l* = −22→22

### Refinement

**Table:**

Refinement on *F*^2^	Primary atom site location: structure-invariant direct methods
Least-squares matrix: full	Secondary atom site location: difference Fourier map
*R*[*F*^2^ > 2σ(*F*^2^)] = 0.044	Hydrogen site location: inferred from neighbouring sites
*wR*(*F*^2^) = 0.113	H-atom parameters constrained
*S* = 1.13	*w* = 1/[σ^2^(*F*_o_^2^) + (0.0561*P*)^2^] where *P* = (*F*_o_^2^ + 2*F*_c_^2^)/3
2486 reflections	(Δ/σ)_max_ < 0.001
310 parameters	Δρ_max_ = 0.12 e Å^−^^3^
6 restraints	Δρ_min_ = −0.18 e Å^−^^3^

### Special details

**Table:**

Geometry. All e.s.d.'s (except the e.s.d. in the dihedral angle between two l.s. planes) are estimated using the full covariance matrix. The cell e.s.d.'s are taken into account individually in the estimation of e.s.d.'s in distances, angles and torsion angles; correlations between e.s.d.'s in cell parameters are only used when they are defined by crystal symmetry. An approximate (isotropic) treatment of cell e.s.d.'s is used for estimating e.s.d.'s involving l.s. planes.
Refinement. Refinement of *F*^2^ against ALL reflections. The weighted *R*-factor *wR* and goodness of fit *S* are based on *F*^2^, conventional *R*-factors *R* are based on *F*, with *F* set to zero for negative *F*^2^. The threshold expression of *F*^2^ > σ(*F*^2^) is used only for calculating *R*-factors(gt) *etc*. and is not relevant to the choice of reflections for refinement. *R*-factors based on *F*^2^ are statistically about twice as large as those based on *F*, and *R*- factors based on ALL data will be even larger.

### Fractional atomic coordinates and isotropic or equivalent isotropic displacement parameters (Å^2^)

**Table:**

	*x*	*y*	*z*	*U*_iso_*/*U*_eq_	
C1	0.1807 (5)	0.6157 (3)	1.2427 (2)	0.0891 (13)	
H1A	0.1266	0.6695	1.2231	0.134*	
H1B	0.2698	0.6414	1.2543	0.134*	
H1C	0.1367	0.5904	1.2835	0.134*	
C2	0.1945 (4)	0.5321 (3)	1.1919 (2)	0.0741 (11)	
H2A	0.2332	0.5582	1.1493	0.089*	
H2B	0.2555	0.4800	1.2098	0.089*	
C3	0.0558 (3)	0.4027 (2)	1.13890 (15)	0.0476 (8)	
C4	0.1641 (3)	0.3628 (2)	1.10191 (16)	0.0554 (9)	
H4	0.2488	0.3953	1.1023	0.066*	
C5	0.1461 (3)	0.2750 (3)	1.06451 (17)	0.0569 (9)	
H5	0.2196	0.2478	1.0402	0.068*	
C6	0.0203 (3)	0.2259 (2)	1.06227 (15)	0.0484 (8)	
C7	−0.0875 (3)	0.2676 (3)	1.09885 (16)	0.0560 (9)	
H7	−0.1727	0.2361	1.0976	0.067*	
C8	−0.0708 (4)	0.3548 (2)	1.13691 (16)	0.0551 (9)	
H8	−0.1442	0.3819	1.1614	0.066*	
C9	−0.2070 (4)	−0.3382 (3)	1.25128 (19)	0.0800 (12)	
H9A	−0.1536	−0.3916	1.2306	0.120*	
H9B	−0.2961	−0.3639	1.2629	0.120*	
H9C	−0.1621	−0.3145	1.2923	0.120*	
C10	−0.2213 (4)	−0.2511 (3)	1.2010 (2)	0.0757 (11)	
H10A	−0.2586	−0.2754	1.1576	0.091*	
H10B	−0.2823	−0.1995	1.2195	0.091*	
C11	−0.0768 (4)	−0.1254 (2)	1.14919 (16)	0.0546 (8)	
C12	0.0506 (4)	−0.0825 (3)	1.14815 (18)	0.0648 (10)	
H12	0.1200	−0.1098	1.1753	0.078*	
C13	0.0772 (4)	0.0010 (3)	1.10721 (17)	0.0584 (9)	
H13	0.1647	0.0290	1.1063	0.070*	
C14	−0.0254 (4)	0.0431 (2)	1.06752 (16)	0.0513 (8)	
C15	−0.1533 (4)	0.0009 (3)	1.06924 (18)	0.0613 (9)	
H15	−0.2233	0.0293	1.0430	0.074*	
C16	−0.1797 (3)	−0.0843 (3)	1.11010 (18)	0.0631 (10)	
H16	−0.2667	−0.1132	1.1108	0.076*	
C17	0.0234 (3)	0.1219 (2)	0.95562 (15)	0.0486 (8)	
C18	0.0227 (3)	0.2085 (2)	0.91266 (15)	0.0494 (8)	
H18	0.0144	0.2732	0.9318	0.059*	
C19	0.0345 (3)	0.1973 (2)	0.84202 (16)	0.0524 (8)	
H19	0.0340	0.2552	0.8142	0.063*	
C20	0.0470 (3)	0.1022 (2)	0.81143 (15)	0.0483 (8)	
C21	0.0489 (4)	0.0180 (2)	0.85418 (17)	0.0556 (9)	
H21	0.0576	−0.0465	0.8348	0.067*	
C22	0.0384 (4)	0.0268 (2)	0.92461 (16)	0.0562 (9)	
H22	0.0413	−0.0314	0.9519	0.067*	
N2	0.0527 (3)	0.0833 (2)	0.73886 (13)	0.0539 (7)	
C24	0.0683 (4)	0.1357 (3)	0.62156 (17)	0.0636 (9)	
C25	0.0822 (7)	0.2158 (4)	0.5769 (2)	0.129 (2)	
H25	0.0939	0.2812	0.5944	0.154*	
C26	0.0793 (8)	0.2014 (4)	0.5064 (2)	0.146 (2)	
H26	0.0878	0.2568	0.4767	0.175*	
C27	0.0637 (7)	0.1046 (4)	0.4803 (2)	0.125 (2)	
H27	0.0633	0.0945	0.4328	0.150*	
C28	0.0490 (5)	0.0243 (3)	0.52299 (19)	0.0818 (12)	
H28	0.0381	−0.0409	0.5050	0.098*	
C29	0.0503 (4)	0.0392 (3)	0.59391 (17)	0.0615 (9)	
N1	0.0028 (3)	0.13169 (19)	1.02609 (13)	0.0572 (8)	
C23	0.0688 (4)	0.1534 (3)	0.69541 (17)	0.0633 (9)	
H23	0.0816	0.2197	0.7113	0.076*	
O1	−0.0896 (3)	−0.21002 (19)	1.19072 (13)	0.0745 (7)	
O2	0.0625 (2)	0.48898 (16)	1.17876 (11)	0.0613 (6)	
O3	0.0333 (4)	−0.04181 (18)	0.63487 (13)	0.0933 (10)	
H3	0.0383	−0.0239	0.6753	0.140*	

### Atomic displacement parameters (Å^2^)

**Table:**

	*U*^11^	*U*^22^	*U*^33^	*U*^12^	*U*^13^	*U*^23^
C1	0.095 (3)	0.078 (3)	0.093 (3)	−0.022 (2)	0.003 (3)	−0.023 (3)
C2	0.069 (3)	0.069 (2)	0.085 (3)	−0.015 (2)	0.005 (2)	−0.012 (2)
C3	0.053 (2)	0.0494 (19)	0.0401 (17)	0.0010 (16)	0.0005 (15)	−0.0007 (15)
C4	0.0502 (18)	0.062 (2)	0.054 (2)	−0.0089 (17)	0.0027 (17)	−0.0064 (19)
C5	0.053 (2)	0.065 (2)	0.0527 (19)	−0.0001 (18)	0.0150 (17)	−0.0058 (18)
C6	0.058 (2)	0.0510 (19)	0.0365 (16)	−0.0029 (17)	0.0009 (15)	0.0031 (15)
C7	0.0462 (19)	0.067 (2)	0.0550 (19)	−0.0081 (17)	0.0033 (16)	0.0012 (19)
C8	0.049 (2)	0.064 (2)	0.052 (2)	0.0052 (17)	0.0050 (16)	−0.0077 (18)
C9	0.097 (3)	0.072 (3)	0.071 (2)	−0.023 (2)	0.002 (2)	0.014 (2)
C10	0.073 (3)	0.072 (2)	0.082 (3)	−0.010 (2)	0.002 (2)	0.008 (2)
C11	0.068 (2)	0.0469 (19)	0.0492 (18)	0.0013 (18)	0.0059 (18)	0.0055 (17)
C12	0.061 (2)	0.067 (2)	0.067 (2)	0.002 (2)	−0.0060 (19)	0.012 (2)
C13	0.054 (2)	0.061 (2)	0.060 (2)	−0.0035 (17)	0.0025 (18)	0.0040 (19)
C14	0.060 (2)	0.0538 (19)	0.0398 (17)	−0.0022 (17)	0.0059 (16)	−0.0005 (16)
C15	0.063 (2)	0.059 (2)	0.062 (2)	−0.0035 (19)	−0.0084 (18)	0.0085 (19)
C16	0.053 (2)	0.063 (2)	0.073 (2)	−0.0079 (18)	0.000 (2)	0.007 (2)
C17	0.0502 (19)	0.056 (2)	0.0397 (17)	−0.0035 (17)	0.0018 (14)	−0.0008 (16)
C18	0.054 (2)	0.0496 (18)	0.0443 (18)	−0.0055 (16)	−0.0008 (15)	−0.0019 (15)
C19	0.055 (2)	0.0566 (19)	0.0460 (18)	−0.0032 (17)	0.0010 (17)	0.0040 (16)
C20	0.0463 (19)	0.059 (2)	0.0395 (16)	0.0027 (17)	−0.0015 (15)	−0.0018 (16)
C21	0.070 (2)	0.0464 (18)	0.0508 (19)	0.0026 (18)	0.0012 (18)	−0.0051 (16)
C22	0.072 (2)	0.0481 (19)	0.0479 (19)	0.0053 (18)	0.0019 (18)	0.0021 (16)
N2	0.0578 (17)	0.0597 (17)	0.0440 (15)	0.0038 (14)	0.0024 (14)	0.0000 (15)
C24	0.081 (2)	0.065 (2)	0.0452 (19)	−0.015 (2)	0.0012 (19)	0.0000 (19)
C25	0.233 (7)	0.091 (3)	0.061 (3)	−0.055 (4)	0.010 (4)	0.006 (3)
C26	0.259 (7)	0.115 (4)	0.063 (3)	−0.054 (4)	0.009 (4)	0.019 (3)
C27	0.208 (6)	0.123 (4)	0.044 (2)	−0.050 (4)	0.001 (3)	−0.006 (3)
C28	0.112 (3)	0.083 (3)	0.051 (2)	−0.011 (3)	−0.009 (2)	−0.007 (2)
C29	0.067 (2)	0.067 (2)	0.051 (2)	0.001 (2)	−0.0048 (19)	0.002 (2)
N1	0.085 (2)	0.0491 (16)	0.0381 (14)	−0.0123 (15)	0.0043 (14)	−0.0009 (13)
C23	0.076 (2)	0.060 (2)	0.053 (2)	−0.014 (2)	0.005 (2)	−0.0069 (19)
O1	0.0706 (17)	0.0684 (15)	0.0846 (17)	−0.0048 (14)	0.0022 (14)	0.0202 (14)
O2	0.0602 (15)	0.0605 (14)	0.0630 (15)	−0.0050 (12)	0.0041 (12)	−0.0160 (12)
O3	0.159 (3)	0.0601 (16)	0.0610 (16)	0.0017 (19)	−0.008 (2)	−0.0043 (14)

### Geometric parameters (Å, º)

**Table:**

C1—C2	1.479 (5)	C14—C15	1.367 (5)
C1—H1A	0.9600	C14—N1	1.439 (4)
C1—H1B	0.9600	C15—C16	1.394 (4)
C1—H1C	0.9600	C15—H15	0.9300
C2—O2	1.431 (4)	C16—H16	0.9300
C2—H2A	0.9700	C17—N1	1.386 (4)
C2—H2B	0.9700	C17—C22	1.392 (4)
C3—O2	1.371 (3)	C17—C18	1.407 (4)
C3—C4	1.380 (4)	C18—C19	1.381 (4)
C3—C8	1.387 (5)	C18—H18	0.9300
C4—C5	1.372 (4)	C19—C20	1.387 (4)
C4—H4	0.9300	C19—H19	0.9300
C5—C6	1.388 (4)	C20—C21	1.380 (4)
C5—H5	0.9300	C20—N2	1.429 (4)
C6—C7	1.382 (4)	C21—C22	1.373 (4)
C6—N1	1.431 (4)	C21—H21	0.9300
C7—C8	1.370 (4)	C22—H22	0.9300
C7—H7	0.9300	N2—C23	1.257 (4)
C8—H8	0.9300	C24—C25	1.368 (5)
C9—C10	1.508 (5)	C24—C29	1.385 (5)
C9—H9A	0.9600	C24—C23	1.450 (5)
C9—H9B	0.9600	C25—C26	1.379 (6)
C9—H9C	0.9600	C25—H25	0.9300
C10—O1	1.408 (4)	C26—C27	1.375 (6)
C10—H10A	0.9700	C26—H26	0.9300
C10—H10B	0.9700	C27—C28	1.347 (6)
C11—C12	1.366 (5)	C27—H27	0.9300
C11—C16	1.369 (5)	C28—C29	1.388 (5)
C11—O1	1.376 (4)	C28—H28	0.9300
C12—C13	1.376 (5)	C29—O3	1.337 (4)
C12—H12	0.9300	C23—H23	0.9300
C13—C14	1.379 (4)	O3—H3	0.8200
C13—H13	0.9300		
			
C2—C1—H1A	109.5	C13—C14—N1	119.8 (3)
C2—C1—H1B	109.5	C14—C15—C16	120.5 (3)
H1A—C1—H1B	109.5	C14—C15—H15	119.7
C2—C1—H1C	109.5	C16—C15—H15	119.7
H1A—C1—H1C	109.5	C11—C16—C15	119.6 (3)
H1B—C1—H1C	109.5	C11—C16—H16	120.2
O2—C2—C1	109.2 (3)	C15—C16—H16	120.2
O2—C2—H2A	109.8	N1—C17—C22	121.5 (3)
C1—C2—H2A	109.8	N1—C17—C18	120.5 (3)
O2—C2—H2B	109.8	C22—C17—C18	117.9 (3)
C1—C2—H2B	109.8	C19—C18—C17	120.0 (3)
H2A—C2—H2B	108.3	C19—C18—H18	120.0
O2—C3—C4	124.7 (3)	C17—C18—H18	120.0
O2—C3—C8	115.6 (3)	C18—C19—C20	121.7 (3)
C4—C3—C8	119.8 (3)	C18—C19—H19	119.1
C5—C4—C3	119.6 (3)	C20—C19—H19	119.1
C5—C4—H4	120.2	C21—C20—C19	117.6 (3)
C3—C4—H4	120.2	C21—C20—N2	116.8 (3)
C4—C5—C6	121.3 (3)	C19—C20—N2	125.5 (3)
C4—C5—H5	119.4	C22—C21—C20	121.9 (3)
C6—C5—H5	119.4	C22—C21—H21	119.1
C7—C6—C5	118.3 (3)	C20—C21—H21	119.1
C7—C6—N1	120.1 (3)	C21—C22—C17	120.8 (3)
C5—C6—N1	121.5 (3)	C21—C22—H22	119.6
C8—C7—C6	121.0 (3)	C17—C22—H22	119.6
C8—C7—H7	119.5	C23—N2—C20	122.5 (3)
C6—C7—H7	119.5	C25—C24—C29	117.9 (3)
C7—C8—C3	119.9 (3)	C25—C24—C23	120.1 (3)
C7—C8—H8	120.0	C29—C24—C23	121.9 (3)
C3—C8—H8	120.0	C24—C25—C26	121.4 (4)
C10—C9—H9A	109.5	C24—C25—H25	119.3
C10—C9—H9B	109.5	C26—C25—H25	119.3
H9A—C9—H9B	109.5	C27—C26—C25	119.5 (4)
C10—C9—H9C	109.5	C27—C26—H26	120.3
H9A—C9—H9C	109.5	C25—C26—H26	120.3
H9B—C9—H9C	109.5	C28—C27—C26	120.5 (4)
O1—C10—C9	107.3 (3)	C28—C27—H27	119.7
O1—C10—H10A	110.3	C26—C27—H27	119.7
C9—C10—H10A	110.3	C27—C28—C29	119.8 (4)
O1—C10—H10B	110.3	C27—C28—H28	120.1
C9—C10—H10B	110.3	C29—C28—H28	120.1
H10A—C10—H10B	108.5	O3—C29—C24	120.8 (3)
C12—C11—C16	119.9 (3)	O3—C29—C28	118.3 (3)
C12—C11—O1	115.1 (3)	C24—C29—C28	120.9 (4)
C16—C11—O1	125.0 (3)	C17—N1—C6	123.0 (3)
C11—C12—C13	120.5 (3)	C17—N1—C14	120.2 (3)
C11—C12—H12	119.7	C6—N1—C14	116.5 (2)
C13—C12—H12	119.7	N2—C23—C24	122.9 (3)
C12—C13—C14	120.2 (3)	N2—C23—H23	118.5
C12—C13—H13	119.9	C24—C23—H23	118.5
C14—C13—H13	119.9	C11—O1—C10	118.3 (3)
C15—C14—C13	119.2 (3)	C3—O2—C2	118.0 (3)
C15—C14—N1	121.0 (3)	C29—O3—H3	109.5
			
O2—C3—C4—C5	179.0 (3)	C29—C24—C25—C26	−0.3 (8)
C8—C3—C4—C5	−1.2 (5)	C23—C24—C25—C26	−178.7 (5)
C3—C4—C5—C6	0.9 (5)	C24—C25—C26—C27	−0.8 (11)
C4—C5—C6—C7	0.0 (5)	C25—C26—C27—C28	1.1 (11)
C4—C5—C6—N1	−176.9 (3)	C26—C27—C28—C29	−0.3 (9)
C5—C6—C7—C8	−0.5 (5)	C25—C24—C29—O3	−178.7 (4)
N1—C6—C7—C8	176.4 (3)	C23—C24—C29—O3	−0.3 (6)
C6—C7—C8—C3	0.2 (5)	C25—C24—C29—C28	1.1 (7)
O2—C3—C8—C7	−179.6 (3)	C23—C24—C29—C28	179.5 (4)
C4—C3—C8—C7	0.7 (5)	C27—C28—C29—O3	178.9 (5)
C16—C11—C12—C13	−1.1 (5)	C27—C28—C29—C24	−0.8 (7)
O1—C11—C12—C13	178.3 (3)	C22—C17—N1—C6	164.1 (3)
C11—C12—C13—C14	1.0 (5)	C18—C17—N1—C6	−19.1 (5)
C12—C13—C14—C15	−0.2 (5)	C22—C17—N1—C14	−9.9 (5)
C12—C13—C14—N1	178.6 (3)	C18—C17—N1—C14	166.8 (3)
C13—C14—C15—C16	−0.6 (5)	C7—C6—N1—C17	121.5 (3)
N1—C14—C15—C16	−179.4 (3)	C5—C6—N1—C17	−61.7 (4)
C12—C11—C16—C15	0.3 (5)	C7—C6—N1—C14	−64.3 (4)
O1—C11—C16—C15	−179.1 (3)	C5—C6—N1—C14	112.6 (4)
C14—C15—C16—C11	0.6 (5)	C15—C14—N1—C17	−79.1 (4)
N1—C17—C18—C19	−175.8 (3)	C13—C14—N1—C17	102.1 (4)
C22—C17—C18—C19	1.1 (5)	C15—C14—N1—C6	106.4 (4)
C17—C18—C19—C20	0.0 (5)	C13—C14—N1—C6	−72.3 (4)
C18—C19—C20—C21	−0.7 (5)	C20—N2—C23—C24	−177.0 (3)
C18—C19—C20—N2	176.7 (3)	C25—C24—C23—N2	178.0 (4)
C19—C20—C21—C22	0.2 (5)	C29—C24—C23—N2	−0.3 (6)
N2—C20—C21—C22	−177.3 (3)	C12—C11—O1—C10	172.5 (3)
C20—C21—C22—C17	0.9 (6)	C16—C11—O1—C10	−8.2 (5)
N1—C17—C22—C21	175.3 (3)	C9—C10—O1—C11	−176.7 (3)
C18—C17—C22—C21	−1.5 (5)	C4—C3—O2—C2	−10.3 (4)
C21—C20—N2—C23	−170.9 (3)	C8—C3—O2—C2	170.0 (3)
C19—C20—N2—C23	11.7 (5)	C1—C2—O2—C3	−172.8 (3)

### Hydrogen-bond geometry (Å, º)

Cg1 Cg2 and Cg3 are the centroids of the C3–C8, C11–C16 and C17–C22 benzene rings, respectively.

**Table:**

*D*—H···*A*	*D*—H	H···*A*	*D*···*A*	*D*—H···*A*
O3—H3···N2	0.82	1.87	2.605 (4)	148
C1—H1*C*···*Cg*2^i^	0.96	2.84	3.760 (4)	162
C4—H4···*Cg*3^ii^	0.93	2.82	3.655 (3)	150
C9—H9*C*···*Cg*1^iii^	0.96	2.94	3.872 (4)	164
C28—H28···*Cg*1^iv^	0.93	2.88	3.742 (4)	154

Symmetry codes: (i) −*x*, *y*+1/2, −*z*+5/2; (ii) *x*+1/2, −*y*+1/2, −*z*+2; (iii) −*x*, *y*−1/2, −*z*+5/2; (iv) −*x*, *y*−1/2, −*z*+3/2.

## References

[bb1] Bruker (2001). *SADABS* Bruker AXS Inc., Madison, Wisconsin, USA.

[bb2] Bruker (2007). *SMART* and *SAINT* Bruker AXS Inc., Madison, Wisconsin, USA.

[bb3] Dharmaraj, N., Viswanathamurthi, P. & Natarajan, K. (2001). *Transition Met. Chem.* **26**, 105–109.

[bb4] Feng, T.-J. (2014). *Acta Cryst.* E**70**, o42. 10.1107/S1600536813033278 PMC3914085 24526988

[bb5] Sheldrick, G. M. (2008). *Acta Cryst.* A**64**, 112–122. 10.1107/S0108767307043930 18156677

[bb6] Tanak, H., Toğurman, F., Kalecik, S., Dege, N. & Yavuz, M. (2013). *Acta Cryst.* E**69**, o1085. 10.1107/S1600536813015407 PMC3770363 24046648

